# Influence of safety warnings on ESA prescribing among dialysis patients using an interrupted time series

**DOI:** 10.1186/1471-2369-14-172

**Published:** 2013-08-09

**Authors:** Mae Thamer, Yi Zhang, Dejian Lai, Onkar Kshirsagar, Dennis Cotter

**Affiliations:** 1 Medical Technology and Practice Patterns Institute, 5272 River Road, Suite 500, Bethesda, MD 20816, USA; 2 University of Texas School of Public Health, 1200 Herman Pressler Street, Suite 1006, Houston, TX 77030, USA; 3 Jiangxi University of Finance and Economics, Nanchang, China

**Keywords:** Epoetin, ESA therapy, Black box warnings, Interrupted time series, Anemia management, ESRD

## Abstract

**Background:**

In March, 2007, a black box warning was issued by the Food and Drug Administration (FDA) to use the lowest possible erythropoiesis-stimulating agents (ESA) doses for treatment of anemia associated with renal disease. The goal is to determine if a change in ESA use was observed following the warning among US dialysis patients.

**Methods:**

ESA therapy was examined from September 2004 through August 2009 (thirty months before and after the FDA black box warning) among adult Medicare hemodialysis patients. An interrupted time series model assessed the impact of the warnings.

**Results:**

The FDA black box warning did not appear to influence ESA prescribing among the overall dialysis population. However, significant declines in ESA therapy after the FDA warnings were observed for selected populations. Patients with a hematocrit ≥36% had a declining month-to-month trend before (−164 units/week, p = <0.0001) and after the warnings (−80 units/week, p = .001), and a large drop in ESA level immediately after the black box (−4,744 units/week, p = <.0001). Not-for-profit facilities had a declining month-to-month trend before the warnings (−90 units/week, p = .009) and a large drop in ESA dose immediately afterwards (−2,487 units/week, p = 0.015). In contrast, for-profit facilities did not have a significant change in ESA prescribing.

**Conclusions:**

ESA therapy had been both profitable for providers and controversial regarding benefits for nearly two decades. The extent to which a FDA black box warning highlighting important safety concerns influenced use of ESA therapy among nephrologists and dialysis providers was unknown. Our study found no evidence of changes in ESA prescribing for the overall dialysis population resulting from a FDA black box warning.

## Background

Anemia affects nearly all end-stage renal disease (ESRD) patients and is associated with reduced quality of life and decreased survival rates [1-3]. In 1987, investigators reported successful use of erythropoietin stimulating agents (ESA also known as rHuEPO, epoetin or EPO, trade name EPOGEN®) in treating the anemia by elevating the hematocrit level of ESRD patients and reducing transfusions. Between 1991 and 2005, the mean ESA dose increased about four-fold in dialysis patients [4]. In 2007, Medicare expenditures for ESAs for ESRD patients were ~ $1.65 billion [5]. Randomized trials found an increase in vascular access thrombosis and a trend towards death and heart attacks among dialysis patients [6], and an increased risk of a composite endpoint including death and cardiovascular events among predialysis patients targeted to higher hematocrit levels [7]. A third study found no difference in harm or benefit in the study arms [8]. As a result, on March 9, 2007, FDA issued a black box public health warning to physicians to adjust the ESA dose to maintain *the lowest hemoglobin level needed to avoid the need for blood transfusions.* According to the FDA, “physicians and patients should carefully weigh the risks of ESAs against transfusion risks.” The goal of this study was to examine if providers changed their prescribing of ESA therapy as a result of the FDA warnings.

## Methods

### Data sources and study design

We used data from the United States Renal Data System (USRDS) Standard Analytic Files (SAFs) to conduct this study [9]. USRDS data system is a national resource that includes demographic and clinical data on ~97% of all US ESRD patients and their institutional providers of dialysis treatment. (The USRDS website, http://www.usrds.org, “Researcher’s Guide to the USRDS Database” describes the variables, data source, collection methods, and validation studies.) The hematocrit reading taken prior to the first administration of ESA therapy during the billing period (usually the beginning of the month) was submitted for payment with the total dose of ESA administered over the exposure period.

Specifically, *September 2004 to February 2007* was chosen as the 30-month base period (prior to the FDA black box warning) and *March 2007 to August 2009* was chosen as the 30-month follow-up period. ESA therapy is usually administered during outpatient dialysis via intravenous administration three times a week. In an effort to stabilize a large increase in hematocrit, physicians will periodically prescribe a zero ESA dose for a particular month. We also include these so-called zero dose months in our analysis. To ensure the availability of claims, the study population was restricted to adult ESRD hemodialysis patients with Medicare as a primary payor indicated by a variable in the USRDS Payor History File. We excluded those patients with MSP because they have incomplete ESA data since their primary (usually private) payor is likely to get billed for ESA therapy. Our study period included only one form of ESA (alfa epoetin) for treatment of anemia associated with renal failure.

Covariates used for subanalyses are listed in Tables 1 &2. Age was categorized as follows: 18 – 44, 45 – 64, and ≥ 65 years. Race was categorized as White or nonwhite. Duration of dialysis was determined as: <12, 12 - <36, and ≥ 36 months. Diabetes was determined if it was reported to be the primary cause of renal failure and/or diabetes was listed as a co-morbid condition when a patient enrolls in the Medicare ESRD program. Dialysis organizational status was defined by: 1) chain membership (based on size and affiliation); and 2) profit status.

**Table 1:** Characteristics of patients receiving ESA therapy before and after the March 2007FDA black box warning

	***Entire study period***	***Base period***	***Follow up***	P-value*
Number of patients months	13,748,238	6,702,212	7,046,026	
	%	%	%	
Age (Year)				*<.0001*
18–44	13.5	13.6	13.4	
45–64	37.9	37.1	38.6	
≥65	48.6	49.3	48.0	
Race				*0.329*
White	54.6	54.6	54.6	
Non-white	45.4	45.4	45.4	
Gender				*<.0001*
Male	53.7	53.4	53.9	
Female	46.3	46.6	46.1	
Duration of dialysis				*<.0001*
<12 months	20.3	21.2	19.5	
12– < 36 months	29.5	29.9	29.2	
≥36 months	50.1	48.9	51.3	
Diabetes co-morbidity				*<.0001*
Diabetic	44.3	44.0	44.6	
Non-diabetic	55.7	56.0	55.4	
Facility profit status				*<.0001*
For-profit	81.2	80.5	82.0	
Non-profit	18.8	19.5	18.0	
Facility chain status				*<.0001*
Chain 1 (FP)	27.0	26.9	27.0	
Chain 2 (FP)	30.4	26.8	33.9	
Chain 3 (NP)	4.0	4.1	3.9	
Medium chain	10.8	13.0	8.8	
Small/non chains	17.8	18.7	16.9	
Hospital-based (NP)	10.0	10.6	9.5	
Hematocrit value^#^				*<.0001*
<30%	8.4	7.4	9.4	
30– < 36%	45.7	41.6	49.8	
≥36%	45.9	51.0	40.8	

Notes: *FP* For-profit, *NP* Nonprofit.

Base period from September 2004 to February 2007. Followup MArch 2007 to August 2009.

FDA black box warning was issuedin March 2007.

^#^Patients months with missing hematocrit values are not included (when dose is withheld).

^*^P-value for Pearson’s chi-square test based on the difference between pre and post FDA warning period.

**Table 2:** ESA prescribing for hemodialysis patients by patients characteristics from September 2004 to August2009

	**Proportion prescribed**	**Average ESA dose/week**			
	**ESA dose**	**Before and after FDA**			
	**≥ 30,000 U/week**	Black box warning *			
	***Overall***	***Base period***		***Followup***	
	***%***	**Mean**	**Std_dev**	**Mean**	**Std_dev**
All	*20.8*	19,486	23,742	18,191	21,581
Age (Year)					
18–44	24.7	21,999	27,397	20,485	24,250
45–64	21.8	20,223	24,668	18,746	22,331
≥ 65	18.8	18,237	21,785	17,106	20,056
Race					
White	19.5	18,427	22,794	17,229	21,008
Non-white	22.3	20,760	24,774	19,350	22,195
Gender					
Male	20.3	18,956	23,937	17,733	21,620
Female	21.3	20,093	23,502	18,729	21,522
Diabetes Co-morbidity					
Diabetic	20.7	19,541	23,223	18,154	21,094
Non-diabetic	20.8	19,443	24,141	18,221	21,964
Facility profit status					
For-profit	22.3	20,943	24,241	19,514	21,927
Non-profit	13.9	13,481	20,496	12,185	18,797
Facility chain status					
Chain 1 (FP)	24.5	23,509	26,658	20,680	22,316
Chain 2 (FP)	22.6	21,071	23,277	20,086	22,043
Medium chains	22.2	20,229	23,350	19,198	22,028
Small/nonchains	17.7	16,850	21,561	15,713	20,029
Hospital-based (NP)	9.9	10,364	19,605	7,700	16,809
Hematocrit value^#^					
< 30%	56.2	40,898	37,024	40,443	31,675
30 – < 36%	24.9	22,807	24,698	21,048	21,596
≥ 36%	13.6	16,383	18,621	13,041	14,971

*Notes:FP* For-profit, *NP* Nonprofit.

Base Period from September 2004 to February 2007. Followup from March 2007 to August 2009.

* P-value based on t-test comparison between base year and followup period within covariate levels was <.0001 for all variables.

# See Footnote from Table 1.

### Interrupted time series analysis

Trends in anemia treatment before and after the FDA Public Health Advisory were statistically analyzed by categorical methods and interrupted time series models [10,11]. To determine if there was a differential impact resulting from the FDA black box warning, we stratified our analyses by demographic, clinical, and facility characteristics. Trends in ESA treatment patterns across the study period were modeled using a general linear model with the monthly dose per week as the dependent variable and the month as the independent variable. Monthly prescription rates for each patient were calculated by dividing the total ESA dose by the number of days in each dialysis claim (typically 30) and multiplying by 7 to calculate the weekly dose per month. These monthly ESA dose per week were then used to determine an average monthly ESA dose per week for the entire population.

An interrupted time series model using the AUTOREG procedure in SAS was used to evaluate changes in average ESA dosages in the 30 months prior (base) and 30 months subsequent (followup) to the FDA black box warning as follows [12]:

$$Y=\mathrm{Bet}a_{0}+\mathrm{Bet}a_{1}*m1+\mathrm{Bet}a_{2}*m2+\mathrm{Bet}a_{3}*x1$$

Where **Y** is the average ESA dose per week in each study month; ***Beta***_***0***_ estimates ESA prescribing at the beginning of the study period; ***Beta***_***1***_ estimates change in ESA prescribing in each month before the FDA warnings (*m1* study months in period 1); ***Beta***_***2***_ estimates change in ESA prescribing in each month after the FDA warnings (*m2* study months in period 2); ***Beta***_***3***_ estimates the change in ESA prescribing level following the FDA warnings (*x1* is an indicator variable (0 for period one and 1 for period two)).

In our model, we took into account *autocorrelations* of the prescribing patterns along the time period. We built our model using a maximum likelihood method with two autocorrelation lags. The first order autocorrelation coefficient was significantly different from zero for almost all models. Time series sometimes exhibit seasonality or seasonal fluctuations. We tested for *seasonality* using proc spectra in SAS against white noise of the residuals from the models and found the residuals from our models were consistent with white noise that indicated that no extra seasonality modeling was needed [13]. In some cases, models need to be corrected for *lagged effects* (i.e., the effect of an intervention might take time to appear). However, in our study, the FDA black box warning was immediately reported to all nephrologists through the Dear Doctor letters, therefore no lag effects were entered into our main model, although possible random lag effects were modeled through serial correlations.

## Results and discussion

### Descriptive statistics

Across the study period, the study population was predominately elderly (49%), white (55%), male (54%), had a duration of dialysis greater than 3 years (50%), were nondiabetic (56%), received dialysis services from for-profit facilities (81%), from one of the two largest for-profit chains (27% and 30%, respectively), and had hematocrit values between 30-36%, within the FDA recommended range (comprising 46% of the study population) or higher (46%) (Table 1).

Although statistically significant, there were only minor differences in patient demographics (age, race and gender), duration of dialysis, and presence of diabetes between the base and followup study periods. While the proportion of patients who received dialysis services in for profit vs. not for profit centers remained nearly the same in the base and followup periods, the largest dialysis chain, Chain 2, appeared to have grown substantially while medium sized chains (defined as chains with 10 to 100 facilities) decreased. There was also a shift in patient hematocrit values between the base and followup study periods; the proportion of patients with values greater than 36% was reduced from 51% to 41%, while those in the 30- < 36% category increased from 42% to 50%. The proportion of anemic patients (<30%) remained ~7-9% during the study period. The aggregate number of patients receiving an ESA prescription (or a zero dose) per month was within the range of 215,444 to 238,697 throughout the study period (data not shown), suggesting no major exogenous change in terms of population size (Table 1).

### Unadjusted analyses and covariate effects

Overall, there was a significant 7% decrease in average ESA dose between the base and followup 30-month periods for all dialysis patients (19,486 versus 18,191 units/week; Table 2). Most covariate strata also showed a decline in average dose between the two periods (*P* < 0.0001), except for Chain 3. Notably, Chain 3 (the largest nonprofit chain), which administers ~4.4% of all ESA doses, administered the lowest mean ESA dose *both* in the base and follow up periods compared to other medium and large dialysis facilities. Overall, nonprofit and hospital-based facilities had the lowest average base and followup ESA doses. Hospital-based facilities are anomalous, however, due to their small size, sicker population, and disproportionate use of darbepoetin, a different form of ESAs that is longer acting was not included in this study.

Younger, nonwhite and new patients were most likely to be prescribed a higher ESA dose (≥ 30,000 units/week, reflecting the highest ESA dose quartile) (*P* < 0.0001). Not unexpectedly, patients with the lowest hematocrit levels were significantly more likely to receive a higher ESA dose (*P* < 0.0001). For profit facilities in general and the two largest for profit chains prescribed higher ESA doses (*P* < 0.0001) (Table 2).

### Interrupted time series results

A model was performed to determine if the average 7% observed decline in ESA dose was consistent with a change in dosing practice as of the FDA black box warning. Model results shown in Table 3 include a general trend in ESA dose/week for each month in the base period prior to the warnings (*Beta*_*1*_), a general trend in ESA dose/week for each month in the followup period after the warnings, often referred to as ‘sustainability’ (*Beta*_*2*_), and a post intervention change (or shift) in ESA dose/week level immediately after the warnings (*Beta*_*3*_).

**Table 3:** Results of interrupted time series model for selected variables

	**Change in ESA/week each month prior to black box warning**			**Change in ESA/week each month post black box warning**			**Change in ESA/eeek Level after black box warning**		
	**Beta1**	**SD**	**p-value**	**Beta2**	**SD**	**p-value**	**Beta3**	**SD**	**p-value**
All	-37	25.8	0.162	-41	27.6	0.146	-882	773.9	0.260
Hematocrit value									
< 30%	224	84.1	0.010	-137	85.7	0.114	6220	2423.0	0.013
30 - < 36%	46	38.1	0.232	-137	85.7	0.114	6220	2423.0	0.013
≥ 36%	-164	23.0	<.0001	-80	23.0	0.001	-4744	648.2	<.0001
Facility profit status									
For-profit	-31	27.7	0.271	-49	30.2	0.109	0.109	835.2	0.440
Non-profit	-90	33.2	0.009	-30	32.7	0.363	-2487	985.4	0.015
Facility chain status ^									
Large chains									
Chain 1 (FP)	-90	30.0	0.004	-103	31.8	0.002	-2148	870.9	0.017
Chain 2 (FP)	-15	35.4	0.673	-27	37.7	0.480	-61	1060.0	0.954
Chain 3 (NP)	94	44.9	0.041	43	44.6	0.343	3189	1288.0	0.016
Medium chains	72	26.2	0.008	-97	26.5	0.001	1703	752.6	0.028
Small/nonchain	-6	24.8	0.797	-49	24.9	0.055	-432	723.3	0.553

*Notes:SD* Standard Deviation, *FP* For-profit, *NP* Nonprofit.

Variables included in Table 3 were selected based on one or more significant Beta value (p < 0.05).

Model intercept coefficient for all variables in Table 1 is significant (p < 0.0001). Since the time after the FDA warnings is not orthogonal to Beta3, the slope after the warnings (Beta2) is conditioned on the change in level (Beta3).

^ Large chains (Chain 1 - Chain 3) are defined as 100 or more units. Medium chains defined as 10 to ≤ 99 units. Small/Nonchains defined as < 10 units.

**Figure 1:**
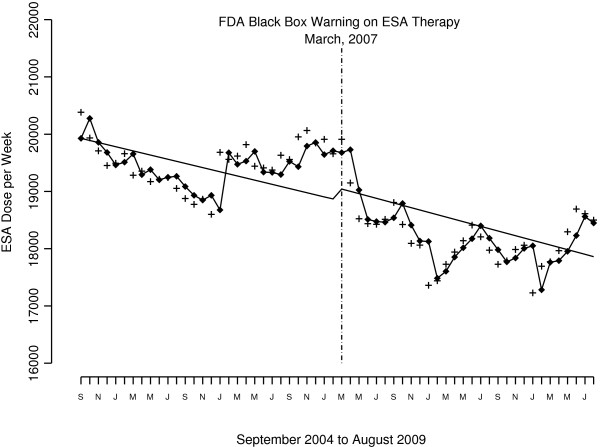
ESA units/week per month for the US hemodialysis population from September 2004 to August 2009.

**Figure 2:**
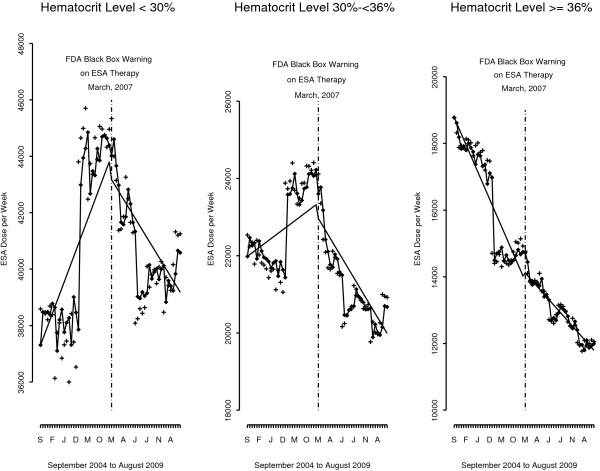
**ESA units/week per month for the US hemodialysis population from September 2004 to August 2009 *by hematocrit level*.** Given the different dosing levels based on hematocrit level, we used a different scale for the Y axis for each hematocrit category, although each covered a range of ~10,000 ESA units.

The FDA black box warning did not appear to influence ESA prescribing among the overall US dialysis population. Model results show the declining trend in month-to-month ESA dose was not statistically significant both before and after the FDA warnings. The drop in ESA dose level after the warnings was also not significant (Figure 1 and first row Table 3). Stratification by patient demographics, clinical and facility characteristics suggest a differential impact in the effect of the FDA black box warning on ESA prescribing. Only a few covariates had statistically significant findings linked to the FDA warnings. For example, patients with a hematocrit ≥36% had a declining month-to-month trend both before (−164 units/week, p = <.0001) and after the warnings (−80 U/wk, p = .001), and a large drop in ESA levels after the warnings (−4,744 U/wk, p = <.0001) (Figures 2 and Table 3**)**. In contrast, patients with a hematocrit <30% had *a large increase* in ESA dose level after the warnings (6,220 U/wk, p = .013), consistent with an *increasing* month-to-month trend before the warnings (224 units/week, p = .01). After the warnings, there was no significant decline in trend in ESA prescribing for patients with a hematocrit <30%. For patients within the FDA-recommended hematocrit range of 30 – 36%, the change in ESA level immediately after the warnings was not significant, but there was a decline in month-to-month trend after the warnings (−103 U/wk, p =0.014).

**Figure 3:**
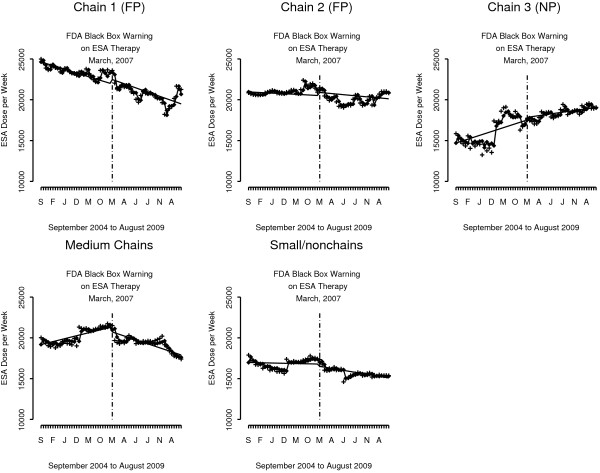
**ESA units/week per month for the US hemodialysis population from September 2004 to August 2009 *by facility organizational status*.** The straight solid line is for the trend derived from the interrupted time series regression model, the cross (+) is the observed value and the diamond (♦) is the predicted value based on the interrupted time series model. The model parameters for all figures are presented in Table 3.

The other area of significant findings and wide variation in response to FDA warnings is dialysis facility organizational status (Figure 3 and Table 3). ESA prescribing trend in not-for profit facilities declined month-to-month before the warnings (−90 units/week, p = .009), with a significant drop in ESA dose immediately after the warnings (−2,487 U/wk, p = <.015). In contrast, there was no evidence of change in ESA prescribing linked to the black box warning among for-profit facilities overall (Table 3). Each of the three largest US dialysis chains responded differently to the FDA warnings. Chain 1 patients experienced a declining month-to-month trend before (−90 units/week, p = .004) and after the warnings (−103 U/wk, p = .002), and a drop in ESA dose level after the warnings (−2,148 U/wk, p = .017). Chain 2 patients experienced no change in ESA prescribing before or after the warnings. And Chain 3 patients experienced an *increasing* month-to-month trend before the warnings (94 U/wk, p = .041), and an *increase* in the ESA dose level after the warnings (3,189 U/wk, p = .016), followed by a flat insignificant trend.

Since ESA dose is sometimes withheld for a particular month as a means of lowering the hematocrit back to the target range, the extent to which dose withholding changed before and after the FDA black box warning is noteworthy. We found only a small increase in the frequency of patient months with zero ESA doses (withholding) in the 30 months after the FDA Black Box warning compared to the 30 months before (12.5% versus 11.5%; data not shown). In addition, we conducted sensitivity analyses including non-zero dose only in the analysis and the results were similar to those reported above. We also used ESA dose per administration (versus dose per week) as the study measure and found similar results. We conducted other models such as a segmented regression approach and the results were very similar to the ITS model reported in the paper.

## Conclusion

Although there was a decline in ESA dose across the 60-month study period, the FDA black box warning issued in March 2007 did not appear to influence ESA prescribing for the overall dialysis population. However, for patients with the highest hematocrit values and for those receiving treatment in certain dialysis facilities, nephrologists and dialysis providers were more likely to heed the FDA black box warning.

The FDA black box warning issued for ESA therapy included the following important study results: “Patients with chronic kidney failure had an increased number of deaths and of non-fatal heart attacks, strokes, heart failure, and blood clots when ESAs were adjusted to maintain higher red blood cell levels (hemoglobin more than 12 g/dL).” A new patient medication guide accompanied the warnings and posed the following question and answer, ‘*What is the most important information I should know about Epogen?* Using Epogen can lead to death or other serious side effects’ [14]. Given these warnings, our findings raise questions as to why providers did not lower ESA doses further than we observed when faced with mounting evidence of risks [7,8,15-18]. Prior to the warnings, and during our study, although the FDA recommended a target hematocrit of 30 – 36%, studies suggested that providers often overshot the high end of this target range given Medicare’s reimbursement policy allowed hematocrit to be as high as 39% [19,20]. ESA therapy was an important source of profit, particularly for large dialysis chains that were also able to recoup large rebates and receive discounts [21], producing the second largest source of facility income of ~22% [22]. For instance, a spike in ESA dose evident in the beginning of 2006, also confirmed by USRDS data [23], appears to be associated with a new lower payment method for ESA therapy that changed from a per-unit rate to a 6 percent above manufacturers’ average sales price (ASP) [24]. During our study period, ESA therapy continued to be reimbursed on a fee-for service basis, creating a financial incentive for increased utilization of this therapy.

Although USRDS data show a decline in both ESA dose and hematocrit levels following the issuance of the FDA black box warning, it remained unclear, until now, whether these results were related to the FDA warnings or rather which groups, if any, benefited from the FDA warnings. Patients who had the highest hematocrit values showed the largest shift or decline in ESA dose level after the FDA warning with a drop of 4,744 U/week, perhaps because providers were concerned about their safety given publication of CHOIR [7] and CREATE [8] findings in mid-November 2006, showing potential harm and no benefit for ESA therapy, respectively. It is noteworthy, however, that *on average* the percentage of patients with a monthly hematocrit reading above 36% declined from 51% to 41%, following the FDA warnings. Given the appropriate goals of ESA therapy, two in five patients had hematocrit levels deemed unacceptably high in the 30 months following the FDA black box warning. For ESA-resistant patients, those with the highest doses and hematocrit levels <30%, there was a large increase in ESA dose level immediately after the warnings (and no subsequent significant decline in ESA trend); findings contrary to the FDA black box warning. Perhaps providers felt justified not to decrease dose for their resistant patients after the warnings given the black box emphasis on avoiding transfusions which are sometimes triggered at a hematocrit threshold of ~27-30% for patients with serious co-morbidities [25]. High hematocrit levels appear to be of more concern to nephrologists than high ESA doses following the black box warnings. Implications of these findings require further investigation.

Variations in treatment practice patterns across more than 4,000 US dialysis facilities are well established and controversial [15,26-31]. In our study, nonprofit facilities overall had a declining trend before the warnings and a large drop in ESA dose immediately afterwards. In contrast, for-profit facilities overall that prescribed *higher* ESA doses in both the period before and after the FDA warnings compared to nonprofit facilities -- on average 19,514 versus 12,185 U/week in the post warning period – did not change their ESA prescribing related to the FDA warnings. However, not all for-profit facilities responded similarly to the FDA warnings. During our study, two-thirds of dialysis patients received treatment in one of two large for-profit dialysis chains. Notably, one chain had significant declines in ESA doses consistent with FDA black box warnings and the other chain did not. Chains are owned by different entities that make individual corporate decisions regarding anemia protocols and anemia management goals among their patients.

Evidence of adverse events commonly emerges after a drug has been on the market for several years necessitating the issuance of a black box warning [32]. According to Green et al. [33], there are three categories of factors relevant to behavior change among physicians: *predisposing factors* (communicating or disseminating information); *enabling factors* (facilitating the desired change in the practice site); and *reinforcing factors* (by reminders or feedback). The model suggests that interventions that are most successful in changing physician practice are those that use enabling strategies or reinforcing methods in addition to predisposing or disseminating strategies. For example, a FDA black box warning on ESA use *for oncology patients* was also released on March 2007, and included a mandate that providers engage in a risk/benefit discussion with the patient and document that this discussion occurred by completing and signing the Patient Acknowledgment Form; a more stringent requirement that is absent from dialysis provider ESA prescribing. In contrast to the results presented herein, ESA use for oncology patients plummeted following the black box warning [34,35].

After nearly three decades of the same ESRD payment system, an enhanced ESRD Prospective Payment System (PPS) was initiated in January 2011 bundling separately billable items (primarily ESA therapy) into the larger dialysis composite rate [36]. Under PPS, facilities have no financial incentive to use more drugs than are clinically necessary. We anticipate that changes in reimbursement rates will have a greater impact on access and reduced exposure to ESA therapy compared to the March 2007 FDA black box warning. Indeed, early indications suggest that both ESA use [37] and hematocrit levels have been dramatically reduced since implementation of ESRD PPS [38].

Several study limitations are noteworthy, however. One, when ESRD patients are hospitalized, on average twice a year, information on ESA dosing is not available. Two, the analysis was confined to those individuals with Medicare as the primary payor, therefore the generalizability to other payers is limited. And three, because of the exploratory nature of our analysis, we did not adjust for the size of type I error rate in conducting multiple statistical tests. One way to address the potential threat regarding the validity of an interrupted time series by historical/secular shifts is to compare, in our case, ESA drug doses to drug dosing of other profitable injectable drugs that should not be affected by the FDA warning (e.g., injectable vitamin D or iron). After increasing each year since 1992 (including growth of 11–19% in 2002–2004) to reach nearly $2 billion, Medicare ESA costs (a surrogate for use) were stable in 2004–2007, and in 2008 declined to a pre-2004 level of $1.8 billion. Conversely, use of other intravenous drugs continued to increase in 2008 — 12% for IV vitamin D, 4.8% for IV iron, and 13.2% for other injectables [39].

ESA therapy had been both profitable for providers and controversial regarding benefits for nearly two decades. The extent to which a FDA black box warning highlighting important safety concerns influenced use of ESA therapy among nephrologists and dialysis providers was unknown. Our study found no evidence of changes in ESA prescribing for the overall dialysis population resulting from a FDA black box warning.

## Competing interests

There are no financial or nonfinancial competing interests for any authors to report; specifically, no authors have any relationships with any companies that may have a financial interest in the information contained in the manuscript. Furthermore, all authors participated in the design, analysis, interpretation, writing, and/or editing of this study and have seen and approved the final version. Dr. Mae Thamer, as principal investigator and first author had full access to all of the data in the study and had final responsibility for the decision to submit for publication.

## Authors’ contributions

MT and DC participated in the design of the study. OK created the analytic files, DL carried out the time series analysis, and MT drafted the manuscript. YZ carefully edited the paper and presentation of results. All authors read and approved the final manuscript.

## Pre-publication history

The pre-publication history for this paper can be accessed here:

http://www.biomedcentral.com/1471-2369/14/172/prepub
